# Survey-based perspectives and reported barriers to *DPYD* testing among U.S. oncologists

**DOI:** 10.3389/fonc.2026.1903512

**Published:** 2026-10-13

**Authors:** Sara Tohme, Ramzi G. Salloum, Jacqueline De La Cruz, Qing Lu, Kejin Dong, J. Kevin Hicks, D. Max Smith, David DeRemer, Pawel Mroz, Sony Tuteja, Larisa H. Cavallari

**Affiliations:** 1 Department of Health Outcomes and Biomedical Informatics, University of Florida, Gainesville, FL, United States; 2 Department of Biostatistics, University of Florida, Gainesville, FL, United States; 3 Departments of Pathology and Translational Pathology, Moffitt Cancer Center, Tampa, FL, United States; 4 MedStar Health, Columbia, MD, United States; 5 Department of Pharmacotherapy and Translational Research and Center for Pharmacogenomics and Precision Medicine, University of Florida, Gainesville, FL, United States; 6 Department of Laboratory Medicine and Pathology, University of Minnesota, Minneapolis, MN, United States; 7 Division of Translational Medicine and Human Genetics, University of Pennsylvania Perelman School of Medicine, Philadelphia, PA, United States

**Keywords:** drug-related side effects and adverse reactions, implementation science, medical oncology, pharmacogenetic testing, precision medicine

## Abstract

**Background:**

Patients with cancer with deleterious *DPYD* variants are at significantly increased risk for severe toxicity with standard fluoropyrimidine doses. Prior to recent guidelines recommending it, the uptake of pre-treatment *DPYD* testing in the United States has been low. We aimed to explore oncologists’ perceptions and barriers to *DPYD* testing adoption on a national level.

**Materials and methods:**

Survey invitations were sent to U.S. oncologists listed in the American Medical Association (AMA) Masterfile in April and May 2025. The surveys assessed physician knowledge, attitudes, and perceptions of *DPYD* testing in clinics caring for patients with cancer in the United States. Descriptive statistics were used to evaluate survey responses.

**Results:**

Of 5,525 physicians invited to participate, 47 oncologists completed the survey (response rate: 0.85%). The majority (85%) indicated that *DPYD* testing could help them manage patients; however, only 34% reported routinely performing *DPYD* testing. Most physicians (77%) agreed that adequate resources were available to order *DPYD* testing. When directly asked, 36% indicated that limited laboratory access was a barrier. In open-ended responses, respondents identified insurance reimbursement concerns (21%) and prolonged turnaround time (19%) as additional barriers. Forty-three percent reported needing assistance interpreting results and applying them to drug selection and dosing. Webinars (25.5%) and self-paced online modules (21%) were the most preferred educational modalities.

**Conclusion:**

Most respondents indicated that *DPYD* testing could help manage patients, yet concerns persist. While regulatory guidance is evolving, these preliminary findings from a small sample highlight the need for future research to characterize implementation barriers and inform policy development.

**Implications for practice:**

Our findings highlight gaps in implementation that can inform targeted strategies to improve the integration of *DPYD* testing into oncology practice, with the potential to enhance patient safety and reduce fluoropyrimidine-related toxicity.

## Introduction

Adverse drug reactions (ADRs) are a leading cause of morbidity and mortality in the United States, with an associated cost exceeding $500 billion/year (1, 2). Approximately 4% of all emergency department visits, and nearly 35% of visits for older adults, are secondary to an adverse drug event (3). In oncology, the prevalence of ADRs is particularly high, with 97% of patients with cancer experiencing ADRs, of which 53% are attributed to cytotoxic anticancer therapies (4, 5). The fluoropyrimidines, 5-fluorouracil (5-FU) and capecitabine, are foundational to the treatment of gastrointestinal cancers including colorectal and pancreatic. Approximately 20% of patients treated with fluoropyrimidine-containing regimens experience early, severe toxicities, including neutropenia, mucositis, severe diarrhea, and hand–foot syndrome, which may lead to treatment interruption, hospitalization, and even death (6, 7).

Dihydropyrimidine dehydrogenase (DPD), encoded by the *DPYD* gene, is the rate-limiting enzyme in fluoropyrimidine metabolism, and DPD deficiency is the primary cause of severe fluoropyrimidine-related toxicity (8, 9). Up to 7% of patients have a *DPYD* variant that causes partial DPD deficiency, and 1 in 1,000 have two deleterious variants resulting in complete deficiency (10). Those with DPD deficiency have a reduced ability to break down fluoropyrimidines to their inactive metabolites, leading to increased exposure to the active cytotoxic drug and a corresponding increased risk for toxicity. However, risk varies substantially depending on the variant’s impact on *DPYD* activity. While severe toxicity and mortality risk are highest in patients with complete enzyme deficiency, heterozygous carriers of decreased-function variants experience lower but still clinically significant toxicity rates with standard doses (11–15). Overall, variant allele carriers experience a 25.6-fold increased risk for treatment-related death compared to wild-type patients (16).

Prospective clinical studies show that *DPYD* testing, with lower fluoropyrimidine doses started in deleterious variant carriers, reduces toxicity and is cost-effective (17–22). Additional data generally suggest comparable overall survival and progression-free survival between patients with deleterious *DPYD* variants treated with reduced fluoropyrimidine doses and noncarriers treated with full doses, although variant-specific differences in progression-free survival have been reported (23–26).

The Clinical Pharmacogenetics Implementation Consortium (CPIC) guideline recommends a 50% fluoropyrimidine dose reduction in patients with one decreased or no-function *DPYD* allele or with two decreased function alleles (i.e., intermediate metabolizers) and avoidance of fluoropyrimidines or a “strongly” reduced dose (e.g., ≥85%) in patients with two no-function alleles or the combination of a no-function and decreased function allele (i.e., poor metabolizers) (10, 11). An exception exists for individuals with the c.1129-5923C>G variant (HapB3 haplotype), for whom the recommendation is to initiate cycle 1 at 75% of the regimen-specific dose based on prescribing guidelines and other patient-specific factors, then escalate toward the regimen-specific dose as tolerated (10). The Food and Drug Administration (FDA) updated the capecitabine and 5-FU labeling in late 2025 and early 2026, respectively, to include boxed warnings about serious life-threatening adverse reactions in patients with DPD deficiency and to advise *DPYD* testing before drug initiation in the absence of an immediate need for treatment (27). In 2020, the European Medicines Agency recommended that patients should be tested before starting treatment with these agents (28). The National Comprehensive Cancer Network (NCCN, v3.2025) recommends discussing *DPYD* genetic testing prior to fluoropyrimidine therapy, with testing decisions individualized based on patient risk assessment. However, the panel acknowledges continuing reservations about routine universal testing (29).

As the clinical applications expand and guidelines are introduced for pharmacogenomic-based diagnostics in cancer therapy, it is important to understand how oncologists perceive this technology, and in particular, their readiness to accept and adopt testing in the clinical setting. Previous investigations have examined knowledge, attitudes, and perceptions towards PGx testing across various healthcare professionals (30–34). While these findings may help to inform implementation at other sites with similar infrastructure in place, most were done in major academic medical centers and focused on institutions with dedicated precision medicine services, limiting generalizability. Hence, the goal of the current survey was to gain a broader understanding of the needs of oncologists in varied settings in the United States, given that they are the ones usually deciding to order testing. Addressing different oncologist perspectives is crucial for healthcare systems to achieve a more comprehensive and sustainable integration of PGx testing.

In this opportune time, we aimed to explore oncologists’ perceptions and barriers to *DPYD* testing uptake and adoption on a national level.

## Materials and methods

### Study design

We conducted a cross-sectional survey with a national sample of oncologists to assess physician knowledge, attitudes, and perceptions of *DPYD* testing in clinics caring for patients with cancer in the United States (see the **Supplementary Material**). This study was reviewed by the University of Florida (UF) Institutional Review Board under Protocol #ET00045374 (approved 4 February 2025) and deemed Exempt Category #2; the data collected posed no risk to subjects.

### Survey content and development

The survey was designed to assess physician knowledge, attitudes, experiences, and perceived barriers related to testing for two pharmacogenes: *DPYD* and *UGT1A1*. Both pharmacogenes are clinically relevant for patients receiving combination chemotherapy regimens for colorectal and other gastrointestinal cancers. To maximize response rates and minimize respondent burden, the survey was subsequently streamlined to focus primarily on *DPYD* by removing most *UGT1A1*-specific questions. However, one Likert-scale question about laboratory access retained the reference to both *DPYD* and *UGT1A1* testing as originally conceived (Question 20). Results reported herein address *DPYD* testing specifically.

### Sampling strategy and recruitment

The survey was administered to physicians (i.e., MDs and DOs) in U.S. oncology practices who cared for patients with cancer. We used the American Medical Association (AMA) Masterfile as our sampling frame and requested a stratified sample of physicians. The Masterfile is a database maintained by the AMA for the purpose of documenting education and professional practice information for nearly all MDs and DOs in the United States. The AMA Masterfile is the most commonly used sampling frame of physicians for health surveys, although physicians can opt out of inclusion. Inclusion criteria required clinicians to be actively practicing in oncology-related specialties, including medical oncology, hematology/oncology, and gynecologic oncology, and engaged in direct patient care. Physicians practicing in office-based, group practice, hospital staff, or teaching settings across multiple U.S. states were eligible. Only physicians with valid email addresses were included in the survey distribution. Physicians who were retired, semi-retired, or inactive were excluded. The final sampling frame consisted of 8,387 eligible physicians meeting these criteria. Respondents were paid $50 remuneration.

### Survey procedures and data collection

We employed a two-phase recruitment strategy to maximize response among oncologists treating patients with fluoropyrimidine-based regimens. In April 2025, 5,131 survey invitations were sent to physicians randomly selected from the AMA Masterfile via Redi-Data using an anonymous survey link. In May 2025, five follow-up reminder emails at different time points were sent via UF Qualtrics to non-respondents from the initial Redi-Data distribution. Additionally, 394 cancer-treating physicians were identified and invited through direct outreach to academic cancer centers and community oncology practices; these physicians were verified to not be duplicates of the original Redi-Data sample. This resulted in a total of 5,525 unique physicians invited to participate. The survey closed in September 2025. Only completed surveys were included in the final analysis. Physicians completed survey questions regarding their attitudes, knowledge, and practices of *DPYD* testing. We did not collect any sensitive information, and all physicians were part of the AMA Masterfile.

### Statistical analysis

Descriptive statistics were calculated for all variables of interest using the *gtsummary* package in R. Continuous variables were summarized using means and standard deviations, and categorical variables were summarized using frequencies and percentages. All survey responses were analyzed using the total respondent sample (*n* = 47) as the denominator.

## Results

Of the 5,525 physicians invited, 98 individuals accessed the survey, and 47 completed it (response rate: 0.85%). Three (6%) were received following the initial invitation, 38 (81%) following reminders, and 6 (13%) through the additional cancer organizations.

Respondent demographics and practice site characteristics are shown in **Table 1**. The majority of respondents held MD degrees. Clinic staffing across respondent sites included a median of 7 physicians, 10 nurses, and 2 pharmacists, with wide variability in staffing levels across sites. A total of 19% of respondents practiced in clinics that were academically affiliated, 34% worked in clinics that were structurally integrated within an academic health system, and 47% reported no academic health system affiliation. Most practice sites were located within close proximity to a major medical center, with 51% situated within 5 miles, whereas 26% were located more than 30 miles away.

**Table 1:** Respondent and practice site characteristics.

Characteristic	Total (*n* = 47)
Degree	
MD	43 (92%)
DO	4 (8%)
Gender	
Male	25 (53%)
Female	19 (41%)
Prefer not to say	3 (6%)
Race	
White	19 (41%)
Asian	16 (34%)
Black or African American	3 (6%)
Two or more races	1 (2%)
Prefer not to say	8 (17%)
Ethnicity	
Non-Hispanic or Latino	39 (83%)
Prefer not to say	8 (17%)
Clinic staffing composition across respondent sites^*^	
Nurses	10 (0–200)
Physicians	7 (1–120)
Pharmacists	2 (0–100)
Nurse practitioners	2 (0–25)
Physician assistants	1 (0–80)
Other clinicians^**^	0 (0–40)
Clinic health system affiliation	
Neither affiliated nor part of an academic health system	22 (47%)
Part of an academic health system	16 (34%)
Affiliated with an academic health system	9 (19%)
Practice site distance from major medical center^***^	
Within 5 miles	24 (51%)
Greater than 30 miles	12 (26%)
Within 11–29 miles	8 (17%)
Within 6–10 miles	3 (6%)

*Clinic staffing variables are presented as median (range) due to non-normal distributions and substantial variability in clinic size.

**Other clinicians included patient counselors, genetic counselors, social workers, palliative care clinicians, pharmacy technicians, and integrative or complementary medicine providers.

***Major medical center defined as the closest center to which clinicians can refer patients for advanced care, including major surgical or inpatient services.

Respondent clinical practice characteristics are shown in **Table 2**. Epic was the most commonly used electronic health record (EHR) system, reported by 58% of respondents. More than two-thirds of physicians indicated either that their EHR lacked a dedicated module for genetic information or that they were unsure of its availability. Physicians reported treating a broad range of cancer types. The most commonly treated malignancies were colorectal and lung cancers (each 79%), followed by liver and gastric cancers (each 74%), pancreatic and prostate cancers (each 72%), and breast and renal cancers (each 70%). A total of 83% (*n* = 39) of responding oncologists had prescribed either capecitabine or 5-FU within the past month; 6% (*n* = 3) had not, and 11% (*n* = 5) did not respond to this item. Regarding clinical practices, 70% of respondents reported having ever ordered any PGx test. Overall, only 34% of respondents reported routinely performing *DPYD* testing. Of those who performed testing, *DPYD* assays were predominantly processed by commercial laboratories (79%), with the remainder conducted in in-house laboratories. The median desired turnaround time for results was 10 days (range: 3–30 days) for respondents with prior testing experience. Physicians who had never ordered *DPYD* testing did not respond to this question, likely because they lacked familiarity with realistic turnaround times or the testing process.

**Table 2:** Oncologist clinical practice characteristics.

Characteristic	Variable	Total (*n* = 47)
Electronic health record system	Epic	27 (58%)
	Cerner	1 (2%)
	MEDITECH	1 (2%)
	Other	18 (38%)
Presence of a dedicated EHR module for genetic information	Yes	15 (32%)
	No	29 (62%)
	Do not know	3 (6%)
Clinical workload	Patients seen per week^*^	78 (46)
Ever ordered any pharmacogenetic test	Yes	33 (70%)
	No	11 (24%)
	Unknown/not sure	3 (6%)
Prescribed capecitabine or 5-fluorouracil in the past month	Yes	39 (83%)
	No	3 (6%)
	Missing	5 (11%)
Frequency of *DPYD* genetic testing	Routinely	16 (34%)
	Occasionally	17 (36%)
	Never	14 (30%)
Cancer types treated by oncologist	Colorectal	37 (79%)
	Lung	37 (79%)
	Liver	35 (74%)
	Stomach (gastric)	35 (74%)
	Pancreatic	34 (72%)
	Prostate	34 (72%)
	Breast	33 (70%)
	Renal	33 (70%)
	Bladder	32 (68%)
	Head and neck	32 (68%)
	Hematologic malignancies	30 (64%)
	Melanoma	30 (64%)
	Gynecological cancers	27 (57%)
	CNS tumors	26 (55%)
	Sarcomas	25 (53%)
	Thyroid	25 (53%)
	Esophageal^**^	1 (2%)
	Ovarian^**^	1 (2%)
	Myelodysplastic syndromes (MDS) ^**^	1 (2%)
	Neuroendocrine tumors^**^	1 (2%)

*This is presented as mean (SD).^**^Cancer types entered as write-in responses.

### Perceived institutional readiness

While the majority of physicians (77%) agreed that *DPYD* testing fit into their current workflow, 11% disagreed or were unsure if it fit. Similarly, the majority of physicians (77%) agreed that adequate resources were available to order *DPYD* testing, whereas 6% reported that such resources were not in place, and 4% were uncertain.

### Identified barriers to testing

Two survey questions assessed barriers to testing. The first asked respondents on a five-point Likert scale whether access to a laboratory that can perform *DPYD* or *UGT1A1* testing was a barrier; 36% strongly agreed or agreed that laboratory access was a barrier. The second question asked respondents in an open-ended format to identify additional barriers to *DPYD* testing. Respondents identified insurance reimbursement concerns (21%), prolonged turnaround time (19%), potential treatment delays (6%), limited clinician education (6%), absence of standardized guidelines (4%), and lack of EHR order sets (4%). These were categorized into operational/workflow, financial, and knowledge/guideline barriers (see **Figure 1**).

**Figure 1:**
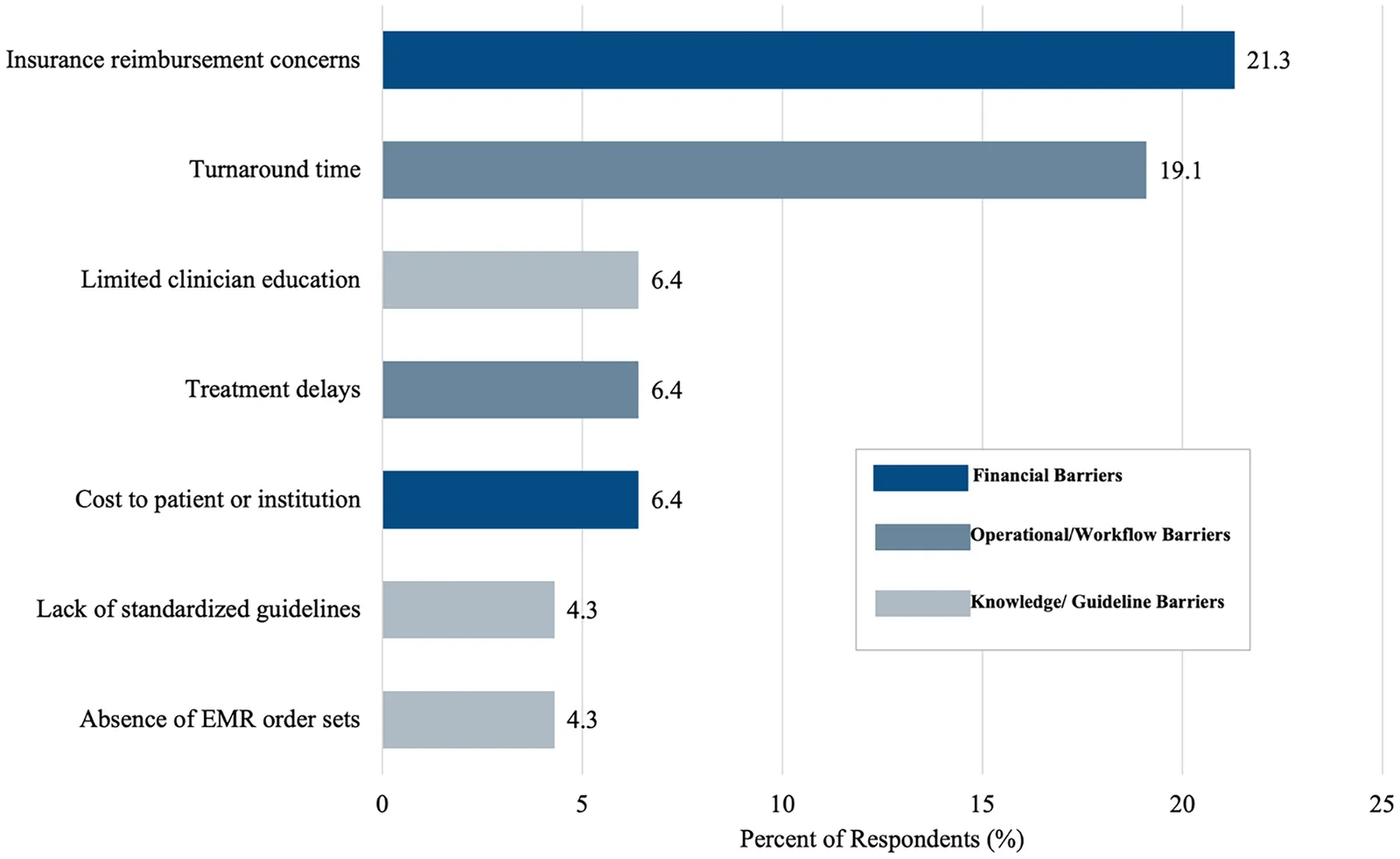
Clinician-reported barriers to *DPYD* testing (*n* = 47).

### Clinician attitudes and preparedness for DPYD-guided therapy

Responses to attitude questions are presented in **Figure 2**. When asked about comfort using *DPYD* test results to adjust patient therapy, 77% answered yes and 23% answered no. When asked about preparedness to discuss *DPYD* testing with patients, 87% answered yes and 13% answered no. Regarding whether *DPYD* testing could help manage patients, 85% answered yes and 15% answered no. When asked whether respondents would need assistance interpreting results, 43% answered yes and 57% answered no. Regarding perceived organizational support, 65% of respondents indicated that senior leadership would support *DPYD* testing implementation (strongly agree or agree), 27% were neutral, and 8% disagreed. Clinicians reported a preference for a variety of educational modalities (see **Figure 3**). Webinars were the most preferred (25.5%), followed by self-paced online modules (21%) and grand rounds or in-house seminars (17%).

**Figure 2:**
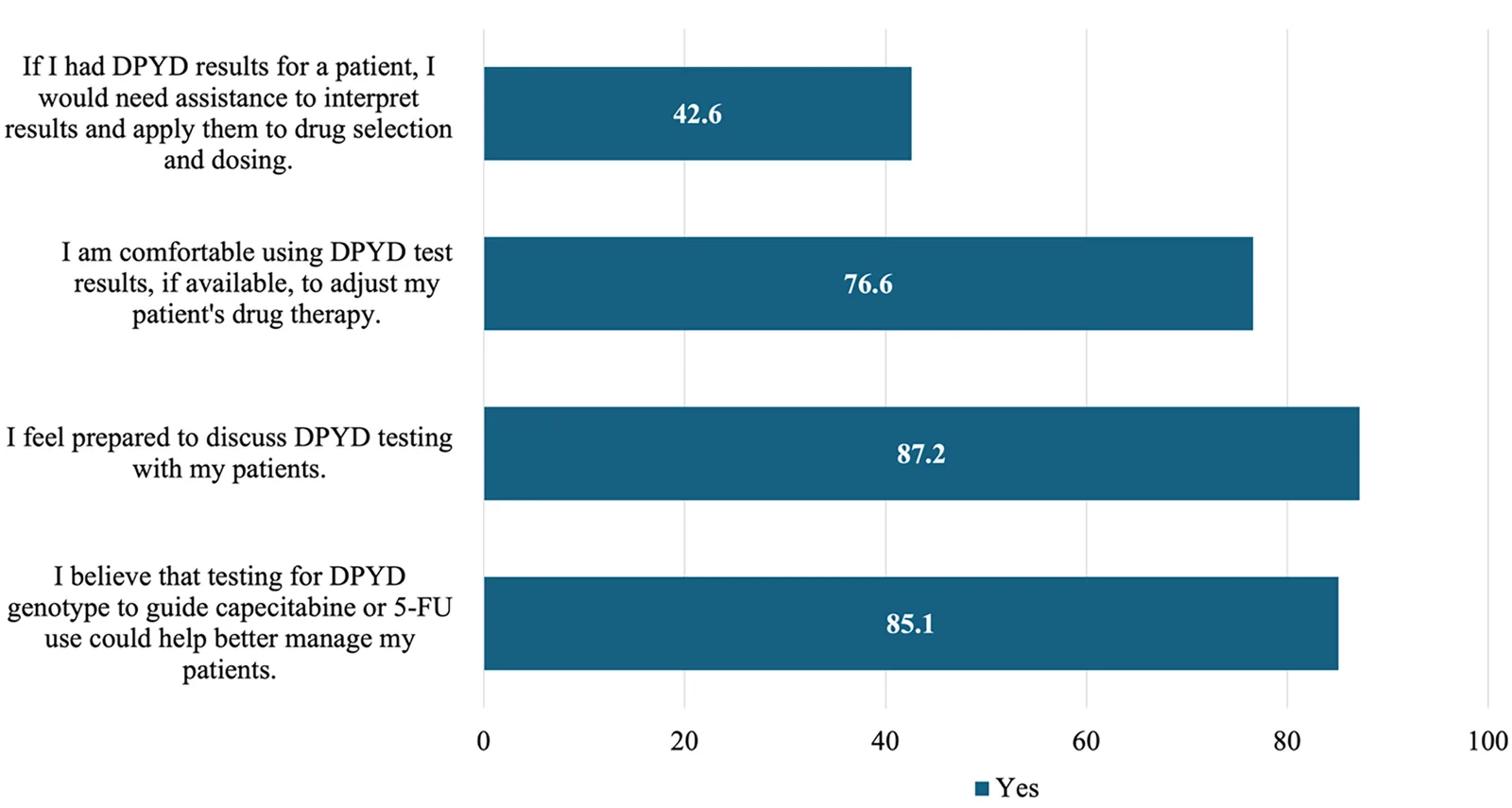
Clinician attitudes and preparedness for *DPYD*-guided therapy (*n* = 47).

**Figure 3:**
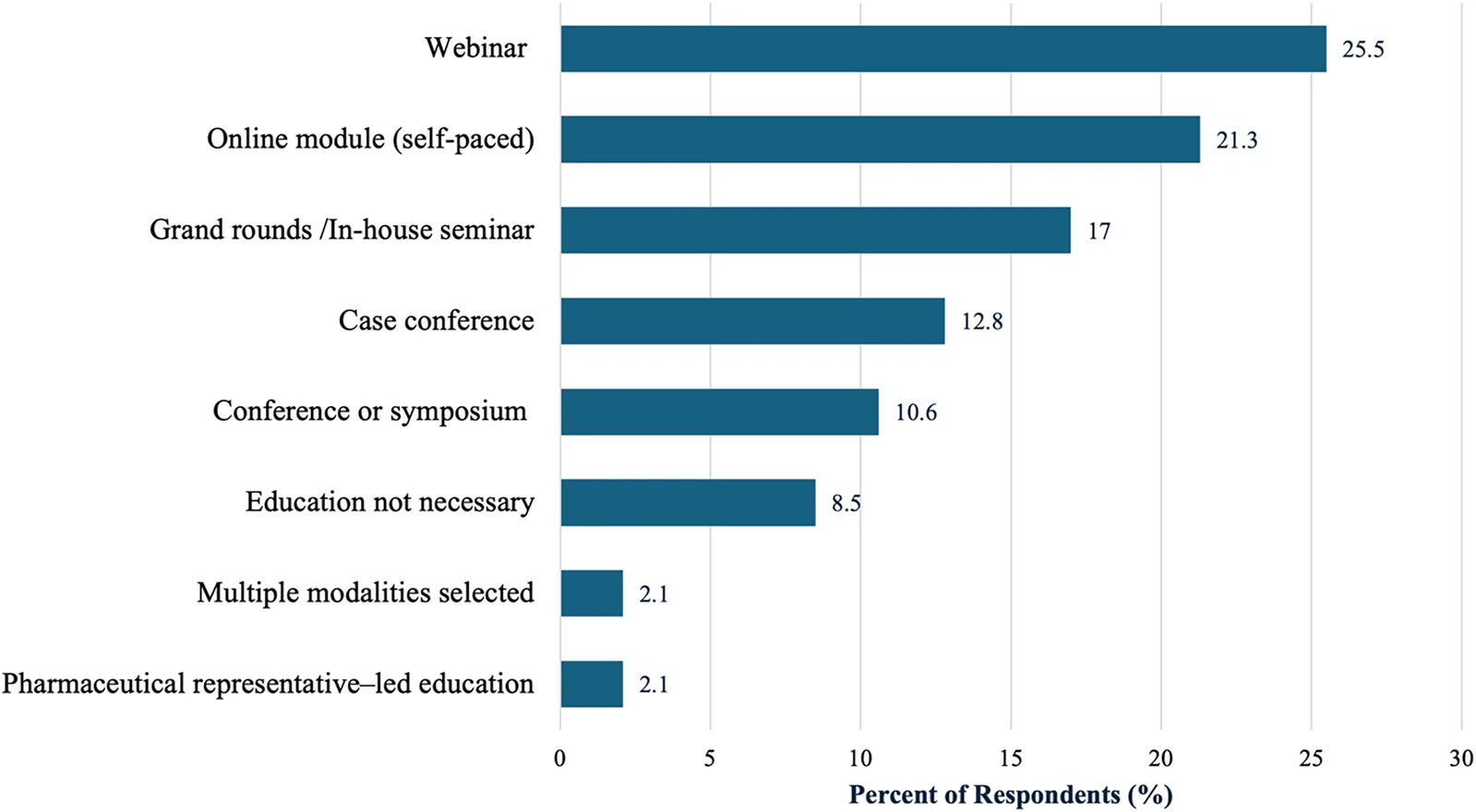
Preferred educational modalities for *DPYD* testing among clinicians (*n* = 47).

## Discussion

There are robust data linking *DPYD* variants to fluoropyrimidine toxicity (11, 12) and evidence of reduced toxicity without an impact on efficacy with *DPYD*-guided fluoropyrimidine dosing (18, 23–26). Genotype-based prescribing guidance is available (11), though revised guidelines incorporating emerging evidence are in development. In Europe, *DPYD* testing became routine after the European Medicines Agency’s 2020 recommendation, which, paired with reimbursement, was followed by rapid increases in pre-treatment testing (9, 35). The FDA did not issue comparable guidance until more than 5 years later, a lag that may reflect differences in health system financing and reimbursement, as well as U.S. guideline reservations about universal testing (29). Even so, *DPYD* testing was increasingly adopted at major U.S. cancer and academic medical centers before the most recent FDA and NCCN guidance (36–39).

Previous surveys suggested that most providers are not prepared for *DPYD* testing implementation (30–34). These investigations have examined knowledge, attitudes, and perceptions towards PGx testing across various provider populations; however, generalizability is limited by the small number of sites involved and their focus on major academic medical centers with dedicated precision medicine services. The goal of the current survey was to gain a broader understanding of the needs and attitudes of oncologists working in varied healthcare settings in the United States.

Among those who responded to our survey, *DPYD* testing uptake was higher than that reported in prior literature. Only 20% of U.S. oncologists reported ever ordering *DPYD* testing, and just 3% reported routine use in a survey administered in 2021 by Koo et al. (30) In contrast, 70% of respondents in our survey reported at least occasionally ordering a *DPYD* test, and 34% reported doing so routinely. Given our small sample size (*n* = 47) and potential overrepresentation of *DPYD*-familiar respondents, this figure might be inflated. However, if it represents a genuine increase in adoption, plausible explanations include evolving evidence over the past few years, the well-publicized FP-related deaths of patients who were not tested for *DPYD* variants, and the lawsuits stemming from these deaths (40, 41). Nonetheless, while the majority of clinicians (85%) in our study indicated that such testing could help manage patients, only 34% reported routine adoption, highlighting a discordance between perceived utility and clinical implementation at the time of survey completion.

Despite generally favorable perceptions of institutional readiness, important workflow-related barriers to *DPYD* testing persist. In our study, 77% of physicians agreed that *DPYD* testing fit within their current workflow and that adequate resources were available; however, a nontrivial proportion reported uncertainty or lack of fit, indicating variability in implementation across settings. When directly asked whether access to laboratories performing *DPYD* or *UGT1A1* testing was a barrier, 36% of physicians strongly agreed or agreed. It is important to contextualize this finding as a potential discrepancy between provider perception and actual availability. A robust infrastructure of both commercial and academic laboratories in the United States currently offers *DPYD* testing (30, 34, 42, 43). The reported lack of access is likely driven by logistical ordering hurdles. Frequently, *DPYD* testing requires a miscellaneous, free-text order, placing the burden on the ordering provider to independently identify and indicate the appropriate laboratory and assay. Therefore, this barrier primarily reflects an integration and workflow challenge rather than a true deficit in testing infrastructure.

Turnaround time emerged as a barrier, with 19% of respondents citing prolonged delays in free-text responses. Among clinicians with prior *DPYD* testing experience, the median desired turnaround time was 10 days (range: 3–30 days), which is theoretically feasible and aligns with commercially available testing timelines. However, this expectation diverges significantly from real-world implementation. El Rouby et al., in a survey of 28 sites that had implemented or planned to implement *DPYD* testing, found that only a minority of test results were returned before fluoropyrimidine therapy initiation (44). This gap between desired and actual turnaround time reflects operational and workflow barriers rather than laboratory limitations (45). To integrate *DPYD* testing into practice, early genetic sampling at the time of cancer diagnosis could help align laboratory turnaround with clinical timing needs. Knowledge and guideline-related barriers were also identified by respondents and remain important contributors to limited *DPYD* testing implementation. In our study, limited clinician education (6%), absence of standardized guidelines at the time of survey distribution (4%), and lack of EHR order sets (4%) were identified as barriers. A primary obstacle is the pervasive lack of comprehensive knowledge among oncologists, which extends beyond unfamiliarity with genetic concepts to include uncertainty in interpreting results and applying them to clinical decision-making (45). Consistent with this, 43% of physicians in our sample reported needing assistance to interpret *DPYD* results and apply them to drug selection and dosing, despite 77% indicating comfort with using results to adjust therapy. Uncertainty also extends to practical aspects of implementation, including how to order tests, integrate workflows for sample collection, and apply results in practice, as noted in prior studies (34, 43). Although most physicians (87%) reported feeling prepared to discuss *DPYD* testing with patients, evolving expectations, such as updated recommendations from the FDA to inform patients about *DPYD*-associated toxicity risks, place additional responsibility on providers and may further expose knowledge gaps. Pharmacists may play a key role in addressing these gaps, particularly in increasing awareness and use of genotype-guided dosing recommendations from the CPIC (10, 11), although pharmacist availability in clinical settings, especially those outside of major medical centers, may be limited. In our study, the median number of pharmacists per site was relatively low compared to other clinical staff, suggesting that limited access to pharmacogenetic expertise may constrain opportunities for clinician education and support in *DPYD* test interpretation and implementation. Clinicians in our study expressed clear preferences for educational interventions, favoring webinars (25.5%), self-paced online modules (21%), and grand rounds or in-house seminars (17%), suggesting that scalable and flexible educational strategies may be effective in improving knowledge and implementation. Collectively, overcoming clinician hesitation requires bridging the gap between test results and actionable interpretation through comprehensive Clinical Decision Support (CDS). Delivering discrete genomic data via the EHR using genomic indicators and Best Practice Advisories (BPAs) is vital, as it alleviates the burden of interpretation and fosters wider clinical acceptance.

In our study, insurance reimbursement concerns were reported as a barrier (21%), and both patient- and institution-level costs were cited as constraints. Prior work by Anantharajah et al. similarly identified cost as a primary patient-reported barrier (46). In addition, inconsistent reimbursement policies and limited economic evidence supporting the cost-effectiveness of PGx testing create uncertainty for implementation (47). However, recent recommendations from NCCN, updates to FDA labeling, and legislation across many states requiring insurer coverage for biomarker testing (31, 36, 44, 48) might improve reimbursement and reduce financial barriers over time.

Several limitations should be considered when interpreting the findings of this study. First, the overall response rate was low, which may limit generalizability. Additionally, response bias is possible, as oncologists with greater familiarity or experience with *DPYD* testing may have been more likely to participate, potentially overrepresenting informed perspectives. To mitigate non-response bias, we employed multiple recruitment strategies; however, these efforts did not substantially increase participation rates. Given the small sample, formal comparison between respondent characteristics and national oncology workforce data was not undertaken. This study surveyed only oncologists; perspectives of pharmacists, laboratory professionals, and regulatory stakeholders, who also shape *DPYD* testing implementation, should be explored in future research. The survey did not assess the timing or indication of *DPYD* testing. Respondents reporting occasional testing may include those ordering testing reactively following fluoropyrimidine toxicity rather than pre-emptively before treatment initiation. Future studies should distinguish pre-emptive from reactive testing. The survey does not ask whether clinicians believe routine *DPYD* testing is clinically appropriate, limiting our ability to distinguish disagreement about testing appropriateness from perceived barriers to implementation. The survey does not assess where clinicians obtain guidance when interpreting abnormal *DPYD* results. Given that 43% reported needing interpretive assistance, understanding actual sources would have been valuable. The item assessing perceived utility (“could help me better manage my patients”) did not specify whether management referred to reducing toxicity, preserving efficacy, or both, limiting interpretation of respondents’ beliefs about the outcomes of *DPYD*-guided dosing. This survey was conducted in April–September 2025, following the NCCN’s April 2025 update recommending discussion of *DPYD* testing but preceding the FDA’s late 2025 and early 2026 updates to drug labeling advising *DPYD* testing before fluoropyrimidine initiation. Findings therefore reflect oncologists’ perspectives and practices during a period of evolving clinical guidance, before widespread implementation of major regulatory changes. As such, the findings may reflect a transitional period and may not fully represent clinical practice following these updates.

## Conclusion

The findings in our study represent the perspectives of a small sample of oncologists and should not be generalized to the broader U.S. oncology community. The low response rate and potential selection bias substantially limit generalizability. Future research with higher participation rates and more representative sampling is needed to definitively characterize implementation barriers and inform policy development.

## Data Availability

The raw data supporting the conclusions of this article will be made available by the authors, without undue reservation.
