# Supplementary material for: Survey-based perspectives and reported barriers to DPYD testing among U.S. oncologists
Source: Front Oncol. 2026 Oct 13;16:1903512. doi: 10.3389/fonc.2026.1903512 (PMC13710826; doi:10.3389/fonc.2026.1903512)
Supplement: Supplementary file 1 [file DataSheet1.pdf]

UF Health
UNIVERSITY OF FLORIDA HEALTH

CLINICIAN SURVEY ON DPYD TESTING

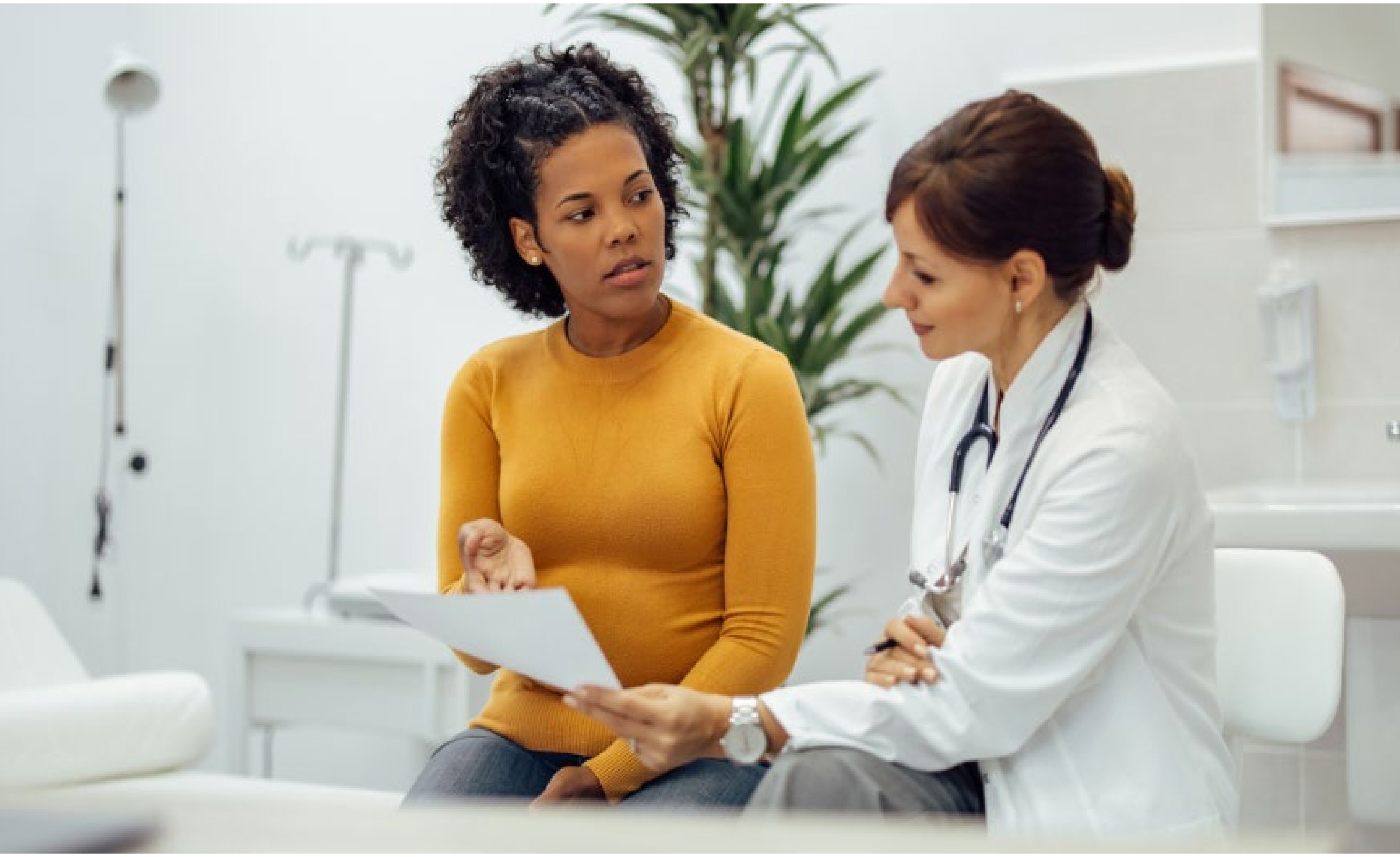

UF Clinical and Translational Science Institute
UNIVERSITY *of* FLORIDA

INFORMED CONSENT FOR CLINICIAN SURVEY

Project Title: Characterizing the Determinants for Pharmacogenetic Testing in Primary Care: A focus on Characterizing the Determinants for DPYD Testing

Principal Investigator: Larisa H. Cavallari, PharmD

1. **Purpose of this study**: The purpose of this research study is to generate representative descriptive statistics on clinician experience with and knowledge, attitudes, and perceptions of DPYD genetic testing in clinics caring for cancer patients in the U.S.
2. **What you will be asked to do**: If you agree to be in this study, you will be asked to complete the following survey. The survey may only be completed once per participant.
3. **Time Required:** The survey should take about 15 minutes to complete.
4. **How many people will be in the study:** 400 people.

   This survey is limited to the first 400 participants who agree to participate in and submit the survey. Once the maximum number of responses is received, the survey will close, and no further entries will be accepted.
5. **If it will cost you anything to take part in this study:** No, there will be no extra cost for you to complete this survey or agree to be in the study.
6. **Participant Compensation:** $50 provided within 2-5 business days. You may select to receive our payment either through Wells Fargo Zelle (delivered via phone/email) or an Amazon e-gift card (email delivery only).

   You are eligible to receive compensation for completing this survey only once.

   If you have any problems regarding your payment, contact the study coordinator, Jacqueline De La Cruz, at (352) 627-9086 or delacruz.j@ufl.edu.

   Your payment for participation in this research study is handled through the University of Florida's Research Participant Payments (RPP) Program. Your information, which will include your name, address, email address, and phone number, is protected. Access to the (RPP) Program site is limited to certain staff with the assigned security role. You will be randomly assigned a specific identification (ID) number to protect your identity.
7. **Risks and Benefits of Participation:** The study has minimal risk. The results may inform resources and tools to assist clinicians with discussing DPYD testing and providing testing and genotype-informed treatment when deemed appropriate.
8. **Statement of Confidentiality:** Your participation in this research is confidential. Information collected about you will be stored in computers with security passwords or in locked filing cabinets. Only certain people have the legal right to review these research records, and they will protect the secrecy (confidentiality) of these records as much as the law allows. These people include the researchers for this study, certain University of Florida officials, and the Institutional Review Board (IRB; an IRB is a group of people who are responsible for looking after the rights and welfare of people taking part in research). Otherwise your research records will not be released without your permission unless required by law or a court order. The researchers will not share your name or other identifiable information about you if they publish, present, or share the results of this research.

   All study data will be entered and collected through an online survey-collection program called Qualtrics. Qualtrics is a secure site with SAS 70 certification for rigorous privacy standards. Any data that you provide through this program will be encrypted for security purposes using Secure Socket Layers (SSL). Only the study investigators will have access to the data on Qualtrics. To protect your privacy, all participants' IP addresses will be masked by Qualtrics and will be unavailable to, and unidentifiable by, investigators or others. Qualtrics' privacy policy can be obtained on the Qualtrics Privacy Statement page.
9. **Voluntary Participation:** Your decision to be in this research is voluntary. You do not have to do any study activities that you do not want to take part in. You can stop at any time. If you decide you want to stop participating in the research, you can let the research team know or call the Principal Investigator at any time at 352-273-8245. If you choose not to take part, this will have no effect on you or your relationships with the University of Florida. If you have any questions about your rights as a research subject, you can phone the Institutional Review Board at 352-273-9600.
10. **Whom to contact if you have questions about the study:**
    Principal Investigator: Larisa H. Cavallari, PharmD, Phone: 352-273-8245
11. **Whom to contact about your rights as a research participant in the study:**
    IRB01 Office, Box 11225, University of Florida, Gainesville, FL 32611-2250; phone (352) 273-9600

SURVEY OVERVIEW

Thank you for participating in this study on DYPD genetic testing to help predict risk for toxicity with fluoropyrimidines: 5-fluorouracil (5-FU) and capecitabine (Xeloda®). Please note that all responses will be kept confidential and that no individual responses will be shared. **This survey should take approximately 15 minutes to complete.**

BACKGROUND

Up to 7% of individuals have a DPYD genotype that increases their risk for potentially life-threatening toxicity, including bone marrow suppression and gastrointestinal toxicity, with 5-FU and capecitabine. NCCN guidelines for GI cancers were recently updated to advise physicians to consider DPYD testing after discussing the risks with patients.

Tests for DPYD variants are commercially available.

The questions in this survey will ask about your background, practice site, and experiences and opinions about DPYD and UGT1A1 testing.

- **Section 1: (13 questions)** Background and Practice Site
- **Section 2: (7 questions)** Experience with DPYD Testing
- **Section 3: (6 questions)** Thoughts on DYPD Testing

BACKGROUND AND PRACTICE SITE
(13 Questions)

1. Number of years in practice: ___________

2. Please select your degree.
   - ☐ MD
   - ☐ DO
   - ☐ Other ________________________

3. What is your gender?
   - ☐ Female
   - ☐ Male
   - ☐ Prefer not to say
   - ☐ Other (Please specify) __________

4. What is your race? (select all that apply)
   - ☐ Native American or Alaska Native
   - ☐ Asian
   - ☐ Black or African American
   - ☐ Native Hawaiian or Other Pacific Islander
   - ☐ White
   - ☐ Two or more races
   - ☐ Prefer not to say

5. Are you of Hispanic, Latino, or Spanish origin?
   - ☐ Hispanic or Latino
   - ☐ Non-Hispanic or Latino
   - ☐ Prefer not to say

6. How many clinicians are in your clinic?

Clinician Type	Number
Physicians	
Physician assistants	
Nurse Practitioners	
Nurses	
Pharmacists	
Other Clinicians	

Please list other clinician types, if applicable:

7. Is your clinic part of, or affiliated with an academic health system?
   - ☐ The clinic is **part of** an academic health system
   - ☐ The clinic **has an affiliation** with an academic health system
   - ☐ The clinic **is neither part of nor affiliated** with an academic health system

8. How far is your primary practice site from the closest major medical center, defined as the closest center you refer patients to for advanced care such as major surgeries or other inpatient services?
   - ☐ Within 5 miles
   - ☐ Within 6-10 miles
   - ☐ Within 11-29 miles
   - ☐ Greater than 30 miles

9. What electronic health record system (EHR) is in place at your site?
   - ☐ Epic
   - ☐ Cerner
   - ☐ MEDITECH
   - ☐ Evident (a CPSI Company)
   - ☐ MEDHOST
   - ☐ Altera Digital Health (a Harris Company)
   - ☐ Vista
   - ☐ Other: ________________________________

10. Do you have a dedicated module in the electronic health record (EHR) to manage genetic information?
    - ☐ Yes
    - ☐ No
    - ☐ Do not know

11. What type of cancer(s) do you treat? Check all that apply.
   - ☐ Bladder
   - ☐ Sarcomas
   - ☐ Breast
   - ☐ Colorectal CNS Tumors
   - ☐ Gynecological cancers
   - ☐ Head and Neck
   - ☐ Hematological cancers
   - ☐ Liver
   - ☐ Lung
   - ☐ Melanoma
   - ☐ Pancreatic
   - ☐ Prostate
   - ☐ Renal
   - ☐ Stomach (gastric) cancer
   - ☐ Thyroid
   - ☐ Other: ______________________________

12. On average, how many patients do you see in one week?

    Please provide a number: _________________

13. Have you ever ordered any pharmacogenetic test (e.g., TPMT, CYP2D6)?
   - ☐ Yes
   - ☐ No
   - ☐ Unknown/not sure

EXPERIENCE WITH DPYD TESTING
(7 Questions)

14. In the past month, I have prescribed Capecitabine or 5-FU
    a. Yes
    b. No

15. How often do you order **DPYD genetic testing** for your patients?
    a. Routinely
    b. Occasionally
    c. Never

16. [If answered "routinely" or "occasionally" above] Who do you order DPYD genetic testing through?
    a. In-house laboratory
    b. Commercial laboratory (e.g., Quest, ARUP)

17. I believe the resources are in place for me to order DPYD testing:
    a. Yes
    b. No
    c. Unknown/not sure

18. If you decided to order DPYD testing for a patient, what is an average test turnaround time needed, in days, for your average patient?

    Please provide number of days: ____________

19. DPYD testing would fit into my current workflow.
    a. Yes
    b. No

20. Access to a laboratory that can perform DPYD or UGT1A1 testing is a barrier.
    a. Strongly agree
    b. Agree
    c. Neutral
    d. Disagree
    e. Strongly disagree

21. What are other barriers to DPYD testing that are not included on this survey? (free response)

THOUGHTS ON DPYD TESTING
(6 Questions)

22. <u>Please indicate 'Yes' or 'No' for the following statements: (Matrix)</u>

	Yes	No
I believe that DPYD testing for fluoropyrimidine toxicity could help me better manage my patients.	O	O
I feel prepared to discuss DPYD testing with my patients	O	O
I am comfortable using DPYD test results, if available, to adjust my patient's drug therapy.	O	O
If I had DPYD test results for a patient, I would need assistance to interpret results and apply them to drug selection and dosing.	O	O

23. Senior leadership/clinical management in my organization would support DPYD testing.

a. Strongly Agree
b. Agree
c. Neutral
d. Disagree
e. Strongly Disagree

24. What would be the <u>most effective method</u> to educate clinicians about DPYD testing?

☐ Case conference
☐ Conference or symposium (half or full day)
☐ Grand rounds or other types of in-house seminars
☐ Online module
☐ Webinar
☐ Other (please specify): ___________________
____
☐ Education is not necessary

Survey Participant Payment Information

Thank you for completing the clinician survey on pharmacogenetic testing!

To receive your compensation for completing this survey, **please complete all fields below**.

All payment methods (Zelle/Amazon) require your first and last name, a valid physical address (no PO boxes), email address, and phone number.

Please provide your full name

First Name: ______________________________________

Last Name: ______________________________________

Valid and verifiable physical U.S. address (no PO boxes)

Street Address: ___________________________________

City: ____

State: _____________

US Zip Code: _______________

Please select a preferred payment method (check one):

Wells Fargo Zelle (email delivery): ________

Wells Fargo Zelle (phone delivery): _______

Amazon e-Gift Card (email delivery only): ________

Email Address & Phone Number (Please provide an email address **and** phone number in which you can receive payment details):

Email: ____

Phone: ____

END OF SURVEY

Thank you for completing the survey!

If you have any questions about the study or payment (if applicable) please contact the study coordinator, Jacqueline De La Cruz, at 352-627-9086 or delacruz.j@ufl.edu
